# Designing System Reforms: Using a Systems Approach to Translate Incident Analyses into Prevention Strategies

**DOI:** 10.3389/fpsyg.2016.01974

**Published:** 2016-12-23

**Authors:** Natassia Goode, Gemma J. M. Read, Michelle R. H. van Mulken, Amanda Clacy, Paul M. Salmon

**Affiliations:** Faculty of Arts, Business and Law, Centre for Human Factors and Sociotechnical Systems, University of the Sunshine CoastMaroochydore, QLD, Australia

**Keywords:** systems thinking, prevention strategies, learning, accidents, accident prevention

## Abstract

Advocates of systems thinking approaches argue that accident prevention strategies should focus on reforming the system rather than on fixing the “broken components.” However, little guidance exists on how organizations can translate incident data into prevention strategies that address the systemic causes of accidents. This article describes and evaluates a series of systems thinking prevention strategies that were designed in response to the analysis of multiple incidents. The study was undertaken in the led outdoor activity (LOA) sector in Australia, which delivers supervised or instructed outdoor activities such as canyoning, sea kayaking, rock climbing and camping. The design process involved workshops with practitioners, and focussed on incident data analyzed using Rasmussen's AcciMap technique. A series of reflection points based on the systemic causes of accidents was used to guide the design process, and the AcciMap technique was used to represent the prevention strategies and the relationships between them, leading to the creation of PreventiMaps. An evaluation of the PreventiMaps revealed that all of them incorporated the core principles of the systems thinking approach and many proposed prevention strategies for improving vertical integration across the LOA system. However, the majority failed to address the migration of work practices and the erosion of risk controls. Overall, the findings suggest that the design process was partially successful in helping practitioners to translate incident data into prevention strategies that addressed the systemic causes of accidents; refinement of the design process is required to focus practitioners more on designing monitoring and feedback mechanisms to support decisions at the higher levels of the system.

## Introduction

Incident reporting and investigation systems are now widely considered to be an essential component of safety management systems, and a pre-requisite for learning from incidents (Nielsen et al., 2006; Lindberg et al., 2010; Jacobsson et al., 2011, 2012). Most organizations have their own reporting and investigation systems; this is a requirement in the international standard for occupational health and safety management (Nielsen et al., 2006). In safety critical domains, such as process control, aviation and healthcare, a number of sector-wide systems have existed since the early 1980s and 2000s (e.g., the Major Accidents Reporting System, 2012; Aviation Safety Reporting System, 2015; and the U.K.'s National Health Service Patient Safety reporting system, Department of Health, 2006). These sector-wide systems are intended to support cross-organizational learning from incidents, as well as reforms to regulation and legislation (Vincent, 2004; Jacobsson et al., 2010; Lindberg et al., 2010). Concerns have been raised, however, that there is little evidence that incident data is actually used to identify prevention strategies or support learning from incidents (Nielsen et al., 2006; Pless, 2008; Jacobsson et al., 2010; Lindberg et al., 2010). One of the reasons underpinning this is the absence of formal processes for translating incident data into appropriate accident prevention strategies^1^. This article describes and evaluates a new process for translating incident data analyses into prevention strategies, based on a systems thinking approach.

### Previous research on translating incident data into prevention strategies

For organizations, “learning from an incident” involves converting an incident experience into activities that will prevent future incidents (Jacobsson's et al., 2012). Several models in the literature describe this process as a series of steps, where no one step can fail without affecting the end result (e.g., Lindberg et al., 2010; Jacobsson's et al., 2012; Drupsteen et al., 2013a). Jacobsson, Ek and Akselsson (2011,?) “learning cycle” model describes the following steps: reporting; analysis; decision-making; implementation; and follow-up. “Reporting” includes the initial reporting and collecting additional data through investigation if required. “Analysis” describes the method for analyzing the data, and designing strategies that prevent similar incidents. “Decision-making” describes the process for selecting prevention strategies for implementation. “Implementation” describes the processes for converting the decisions into action. Finally, “Follow-up” includes both monitoring the implementation, and evaluating the impact of the action.

The majority of research examining aspects of the learning cycle has focused on the methods used to investigate incidents and analyze the data (for a review see Katsakiori et al., 2009). In addition, there is a significant body of research examining the factors influencing initial reporting, and the selection, implementation and maintenance of prevention strategies (e.g., Pidgeon and O'Leary, 2000; Lundberg et al., 2010, 2012; Ramanujam and Goodman, 2011; Le Coze, 2013; Vastveit et al., 2015). However, little research has focused on the process of designing prevention strategies, or describing the prevention strategies that result.

This lack of research into the design of prevention strategies implies that there is a belief that the analysis of incident data will *automatically* lead to new knowledge, new structures, new rules, and new practices that will result in higher reliability and improved safety once implemented (Lundberg et al., 2010; Carroll and Fahlbruch, 2011; Drupsteen et al., 2013b). However, examinations of investigation manuals show that little guidance is provided on how to design prevention strategies based on the outputs from an investigation (Lundberg et al., 2009; Rollenhagen et al., 2010; Drupsteen et al., 2013b). It is therefore unclear how safety practitioners design prevention strategies from the causes that are found, or prioritize addressing certain causes over others.

Another issue is that investigation manuals often give little consideration to understanding how the implementation of specific prevention strategies might impact on the system as a whole (Johnson, 2003; Lundberg et al., 2009; Rollenhagen et al., 2010). The approach to developing prevention strategies in many organizations is to address each cause identified in isolation (Johnson, 2003; Lundberg et al., 2009; Drupsteen and Hasle, 2014). This is problematic as changes to any system component will necessarily impact on others, and potentially lead to unintended, negative consequences (Lundberg et al., 2009; Kirwan, 2011). One reason for this may be that many investigations are still underpinned by linear chain-of-event accident causation models. These models focus safety practitioners on the negative events within an accident sequences and the “broken” components of the system. The underlying accident model therefore works against understanding the system as a whole (Lundberg et al., 2009; Rollenhagen et al., 2010; Dekker, 2011; Leveson, 2011).

A number of authors have argued that using a systems-based accident causation model to collect and analyze incident data might better support addressing problems holistically, rather than just treating individual parts of the system (Dekker, 2011; Leveson, 2011; Hollnagel, 2012). Systemic models are underpinned by three core principles of accident causation. First, safety in work systems is impacted by decisions and actions made at all levels of the system, not just by human operators working within the immediate context of the hazardous processes. Second, accidents are caused by multiple factors that go beyond the immediate context of the incident. Third, accidents and safety are described as emergent properties of systems, arising from interactions between the components within that system (Hollnagel, 2004; Leveson, 2011). Accidents and safety are considered to be “emergent properties” as the outcome of interactions between the components cannot be predicted from examining the functioning or reliability of each components in isolation (Dekker et al., 2011; Leveson, 2011). Based on these principles, it has been argued that prevention strategies should focus on addressing the factors at the higher levels of the system that create hazardous conditions and unsafe acts, rather than directly on failures relating to technology or human operators (e.g., Rasmussen, 1997; Dekker, 2011). In addition, it is the authors' opinion that these principles imply that organizations need to identify networks of prevention strategies, rather than standalone ones, in order to address failures arising from interactions between the components in the system.

A number of systems-based analysis methods have been developed that represent the contributing factors involved in accidents as complex, non-linear causal networks (e.g., STAMP, Leveson, 2011; AcciMap, Rasmussen, 1997). Many studies have demonstrated that they provide a deeper understanding of how interactions within systems contribute to hazardous conditions and unsafe behavior in a range of safety-critical domains including space exploration (Johnson and Muniz de Almeida, 2008), aviation (Branford, 2011), rail (Underwood and Waterson, 2014), public health (Cassano-Piche et al., 2009), disaster management (Salmon et al., 2014a), road freight transport (Salmon et al., 2013; Newnam and Goode, 2015), and led outdoor activities (Salmon et al., 2014b, 2016a). Although these studies have focused on describing how accidents are caused, rather than how they can be prevented, there is no obvious reason why the same methods could not be applied to both analyze accidents and identify prevention strategies (Salmon et al., 2016b). Potentially, these methods could be extended to provide a structured process for translating incident data analyses into prevention strategies. If this approach is successful, the resulting prevention strategies should address the systemic causes of accidents.

This article investigates this proposition further by presenting the findings from a study using a systems approach to accident analysis and the prevention strategy design process. The study involved conducting participatory workshops with practitioners to identify prevention strategies from incident data collected through a national reporting system from the led outdoor activity (LOA) sector in Australia. The collection and analysis of the incident data, and the workshop prevention strategy design process, were all based on Rasmussen's (1997) risk management framework and associated AcciMap technique. The following sections provide a brief overview of both, along with details of their application to the LOA sector and the current study.

### Rasmussen's risk management framework and AcciMap

**Figure 1:**
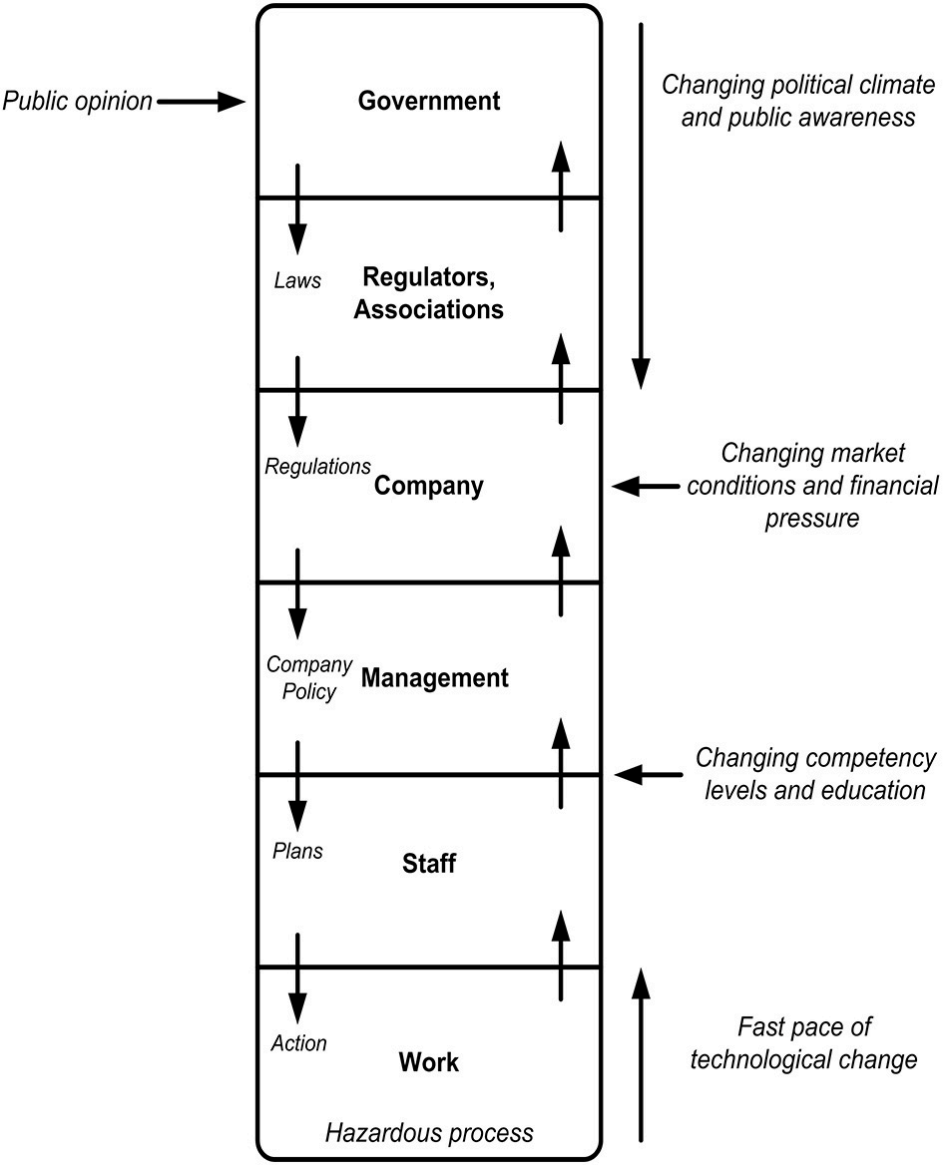
**Rasmussen's risk management framework (adapted from Rasmussen, 1997)**.

Rasmussen's (1997) risk management framework is underpinned by the idea that work systems can be described as a hierarchy of multiple levels (e.g., government, regulators/associations, company, management, staff, work), as shown in Figure 1. The actions and decisions of those operating within and across these levels interact, and contribute to the control of hazardous processes. Safety is maintained through a process referred to as “vertical integration,” where decisions made at higher levels of the system (i.e., by government, regulators, and the company) are reflected in practices occurring at lower levels of the system, while information at lower levels (i.e., work, staff) informs decisions and actions at the higher levels of the hierarchy. A lack of vertical integration can result in a loss of control and accidents (Svedung and Rasmussen, 2002; Cassano-Piche et al., 2009). The framework also describes how work practices constantly adapt and change in response to various external pressures and conditions. This process, referred to as “migration,” causes accidents when changes in work practices erode existing control measures (Rasmussen, 1997).

The accompanying AcciMap technique provides a methodological framework for analyzing accidents from this perspective. The method enables analysts to graphically represent the contributing factors across all levels of the system in question, along with the relationships between them (Rasmussen, 1997; Svedung and Rasmussen, 2002).

Rasmussen's framework also makes a series of predictions, shown in Table 1, regarding accidents and safety in complex sociotechnical systems. These predictions reflect the three core principles of accident causation underpinning the systems approach, and also describe the role that vertical integration and the migration of work practices play in accident causation. These predictions have been used to evaluate the applicability of Rasmussen's framework and the AcciMap technique in new domains (e.g., Cassano-Piche et al., 2009; Jenkins et al., 2010; Salmon et al., 2014a), and to evaluate whether accident investigation processes adequately support the application of systems analysis methods (Newnam and Goode, 2015).

**Table 1:** **Rasmussen's predictions regarding performance and safety in complex sociotechnical systems**.

1.	Safety is an emergent property—it is impacted by the decisions of all actors within the system
2.	Accidents are caused by multiple contributing factors, not a single catastrophic decision or action
3.	Accidents can result from a lack of vertical integration across levels, not just deficiencies at any one level alone
4.	Lack of vertical integration is caused by a lack of feedback across levels. Actors cannot see how their decisions interact with those made by actors at other levels so threats to safety are not obvious before an accident
5.	Work practices are not static, they migrate over time under the influence of a cost gradient driven by financial pressures in an aggressive competitive environment and under the influence of an effort gradient driven by the psychological pressure to follow the path of least resistance
6.	Migration of work practices can occur at multiple levels, not just in one level alone
7.	Migration of work practices causes the system's defenses to degrade and erode gradually over time

In the current study, the AcciMap technique was used initially to graphically represent the contributing factors, and the relationships between them, which were identified from incidents reported in the LOA sector in Australia. It was also subsequently used to represent networks of prevention strategies proposed to address these contributing factors and prevent future occurrences of similar incidents. Rasmussen's predictions were used to underpin the prevention strategy design process, and to evaluate whether the resulting prevention strategies address the systemic causes of accidents. These applications are described in detail in the following sections.

### Application to incident data collection and analysis in the LOA sector

The research described in this article was undertaken in the LOA sector in Australia. This sector includes all organizations that facilitate supervised or instructed “led” outdoor activities, such as outdoor education and recreation providers, school camps, adventure tourism operators and outdoor therapy programs (Goode et al., 2014a). These organizations deliver potentially high-risk activities (e.g., canyoning, sea kayaking, rock climbing, camping) in dynamic environments.

In the past 10 years, a number of high profile fatalities have occurred in Australia and internationally, which highlighted the need for better methods for understanding and preventing incidents in this domain (Salmon et al., 2010, 2012). For example, six students and their teacher died while on a gorge walking activity in New Zealand in 2008. The coroner and an independent investigation highlighted multiple contributing factors relating to the instructor, her manager, the activity center, the local weather service and government legislation and regulation (Brookes et al., 2009; Davenport, 2010). Previous literature on incident causation in this domain had focused on the immediate context of the incident (e.g., activity leader knowledge of environmental hazards and experience, supervision, weather), with little acknowledgement of the factors at the higher levels of the system (e.g., Curtis, 1995; Brookes, 2003, 2004).

There is now significant evidence that accident analysis methods underpinned by a systems approach are required to understand the incidents that occur during led outdoor activities. Analyses of fatal incidents (Salmon et al., 2010, 2012), near misses, and more common everyday injuries and illnesses (Salmon et al., 2014b, 2016a) have identified multiple contributing factors. In this domain, illnesses are viewed as important as even relatively minor illnesses or allergies may pose a serious risk in remote or wilderness environments (Goode et al., 2015).

To support the collection of incident data in the Australian LOA sector from a systems perspective, the authors have used Rasmussen's (1997) risk management framework to underpin the development of a national incident reporting system (Goode et al., 2015; Salmon et al., 2016a). The Understanding and Preventing Led Outdoor Accidents Data System (UPLOADS) allows organizations to record detailed information on incidents, including the event itself (e.g., the activity, the participants and supervisory staff involved), relevant events leading up the incident, and describe the system of contributing factors that staff and management perceive to be involved. This data is then sent to the research team for analysis, and reports are produced annually.

**Figure 2:**
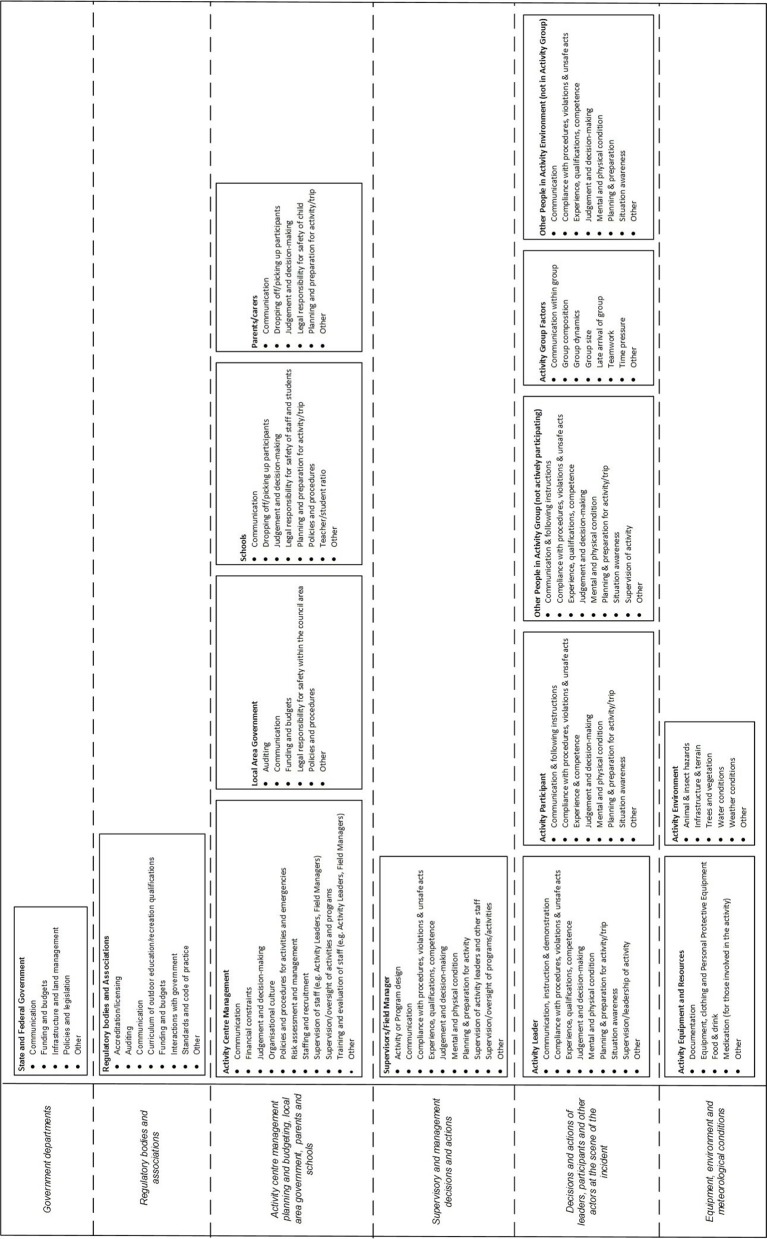
**LOA contributing factor classification scheme based on Rasmussen's framework and AcciMap technique**.

To standardize the analysis of the incident data by the research team, the authors have developed a domain-specific contributing factor classification scheme, based on Rasmussen's framework and AcciMap technique. The classification scheme, shown in Figure 2, describes the actors and contributing factors involved in incidents across the LOA system. The classification scheme was developed and refined in a series of previous studies (Goode et al., 2014b; Salmon et al., 2014b; Taylor et al., 2015a,b).

Injury, illness and near miss incident data reported and analyzed via UPLOADS over a 12 month period (1st June 2014—31st May 2015) were used as the primary source of information for the prevention strategy development workshop. The prevention strategy design process focused on three AcciMaps representing the contributing factors identified from the injury, illness and near miss data. Due to space restrictions, only the prevention strategies relating to the injury data are presented in this paper.

### Application to the prevention strategy design process in this study

Rasmussen's framework and the AcciMap technique were also used to underpin the prevention strategy design process. During the design process, the AcciMaps representing the incident data were used to identify specific goals for incident prevention. For each specific goal, a network of prevention strategies, and the potential relationships between them, were identified. Each prevention strategy identified a specific action and the actors that would be responsible for implementation. Relationships between the prevention strategies were used to describe how the successful implementation of one prevention strategy depended on another, or how the prevention strategies supported better vertical integration. The prevention strategies and the relationships between them were mapped onto the framework shown in Figure 2 using the AcciMap technique (the resulting diagrams are referred to as PreventiMaps in this paper).

To guide the prevention strategy design process, Rasmussen's predictions were used to derive a series of reflection points (see Table 2). These reflection points were used by workshop facilitators to prompt practitioners to think about the incident data and prevention strategies from a systems perspective. In addition, a key question for this article was whether this design process resulted in prevention strategies that addressed the systemic causes of accidents. Therefore, Rasmussen's predictions were also used to develop criteria for evaluating the networks of prevention strategies developed during the workshops (see Table 2).

**Table 2:** **Reflection points developed for the prevention strategy design process and the criteria used to evaluate the resulting PreventiMaps based on Rasmussen's predictions**.

	**Reflection points**	**Evaluation criteria**
1	Can you see in the AcciMap how decisions and interactions between actors created situations where incidents occurred?	The prevention strategies require actions and decisions from multiple actors (at least three).
	This solution relates to one actor, can you think of related solutions that fit in other levels of the AcciMap structure?	The prevention strategies require changes at multiple levels of the system (at least three).
	How does the solution support interaction/coordination across actors at different levels?	
2	Is there an obvious set of contributing factors in the AcciMap that appears to be important?	Multiple interdependent prevention strategies are identified to address the specified goal (at least three). These include mechanisms to support the implementation of prevention strategies within and across levels.
	Could this solution be part of a wider set—what is needed at the level above to make it work? What is needed at the level below?	
3	Can we improve communication and coordination across the levels to improve this issue?	The prevention strategies support the flow of information from actors across and within system levels.
	Could information flowing upwards be improved?	
	Could information flowing downwards be improved?	
	Could information flow within actors at the same level be improved?	
	To make this idea work what would need to be communicated up to the higher levels? What would need to be communicated down to the lower levels?	
	To make this solution work, how does information need to flow between actors—upwards, downwards, and across levels of the system?	
4	Can we improve feedback across levels of the system so that an actor knows the outcomes of their decisions and actions?	The prevention strategies improve feedback processes to actors regarding the impact of their decisions and actions.
5	How might financial pressures impact on this solution, especially over time? Is it financially sustainable? Can we improve this?	The prevention strategies provide mechanisms for actors at the higher levels to identify or monitor changes to work practices at the frontline of operation.
	How might psychological pressures impact on this solution, especially over time? Will people see its ongoing relevance? Can we improve this?	
	How could we identify or monitor changes to work practices as a result of financial pressures or psychological pressures?	
6	How might financial pressures at a higher/lower level of the system impact on this solution?	The prevention strategies provide mechanisms for monitoring changes to work practices for actors at the higher levels of the system.
	How might psychological pressures at a higher/lower level of the system impact on this solution?	
7	How could we monitor whether defenses are degrading/eroding over time within organizations and/or across the sector?	The prevention strategies include mechanisms for monitoring whether the implementation of risk control measures are degrading over time.

*Numbers relate to the predictions shown in Table 1*.

In summary, the aims of this article are to: (1) describe the prevention strategies that were developed using a systems thinking approach; and (2) evaluate the extent to which they address the systemic causes of accidents as defined by Rasmussen's risk management framework.

## Methods

### Design

Two workshops with practitioners from the LOA sector in Australia were conducted to design prevention strategies based on incident data. Ethics approval was obtained from the University of the Sunshine Coast Human Research Ethics Committee.

### Participants

Participants were invited to workshops based on their experience and role within the sector, or role in regulating safety within the sector. The aim was to ensure that the workshops included representatives from across the LOA system, including actors from the following: secondary schools; outdoor education providers; outdoor training organizations; outdoor sector Peak bodies; work health and safety (WHS) regulator; and relevant government departments.

**Figure 3:**
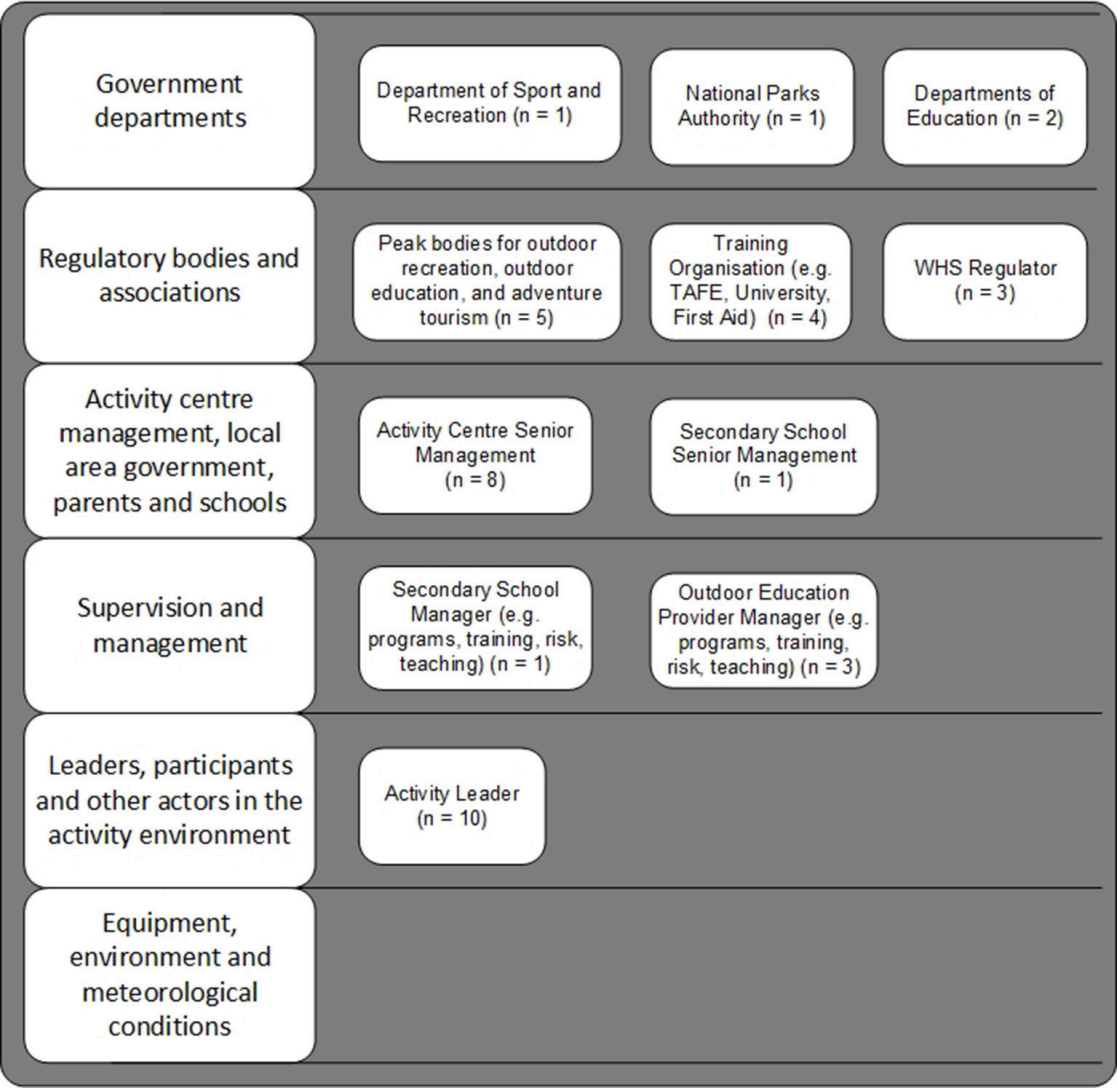
**Number of workshop participants representing each actor within the sector**. Ten participants were Activity Leaders in addition to holding managerial roles within their organization. In relation to the “Equipment, environment and meteorological conditions level,” Senior Managers would purchase equipment, Field Managers would ensure equipment maintenance and serviceability, and Activity Leaders would use the equipment.

In total, 30 people attended the workshops (Workshop 1 = 20, Workshop 2 = 10). The majority of participants were male (25 males, 5 females) and had a mean age of 47 years (*SD* = 9.53), with a mean of 21 years' experience in the outdoor sector (*SD* = 9.52, missing = 3). The number of workshop participants representing each actor within the sector is represented in Figure 3 (note that some participants held more than one role).

### Workshop planning activities

Materials from a systems thinking-based design toolkit (Read et al., 2015), originally developed for use with the Cognitive Work Analysis (CWA) framework (Vicente, 1999), were adapted for use with the AcciMap analyses. The toolkit provides a structured approach for translating the outcomes of systems analysis methods into design concepts. The toolkit provided guidance on who should participate in the workshops and the type of group discussion activities required during the design process. Applying the toolkit resulted in a workshop plan and a set of reflection points to guide the design process based on Rasmussen's predictions (see Table 2).

### Materials

#### Incident data and analysis

The incident data was collected over a 12-month period (1st June 2014—31st May 2015) by 31 LOA organizations across Australia. The organizations used UPLOADS to collect information about the injuries, illnesses and near misses that occurred during LOA programs during this period. Injuries and illnesses were defined as any issue that required care. This included any injury or illness requiring localized care with short term effects through to fatalities. A near miss was defined as “as a serious error or mishap that has the potential to cause an adverse event but fails to do so because of chance or because it is intercepted. For example, during a rock climbing activity an instructor notices that a participant's carabineer was not locked. If the student had fallen, this may have led to a serious injury.” The organizations submitted deidentified data to the research team on a quarterly basis (van Mulken et al., 2016).

**Figure 4:**
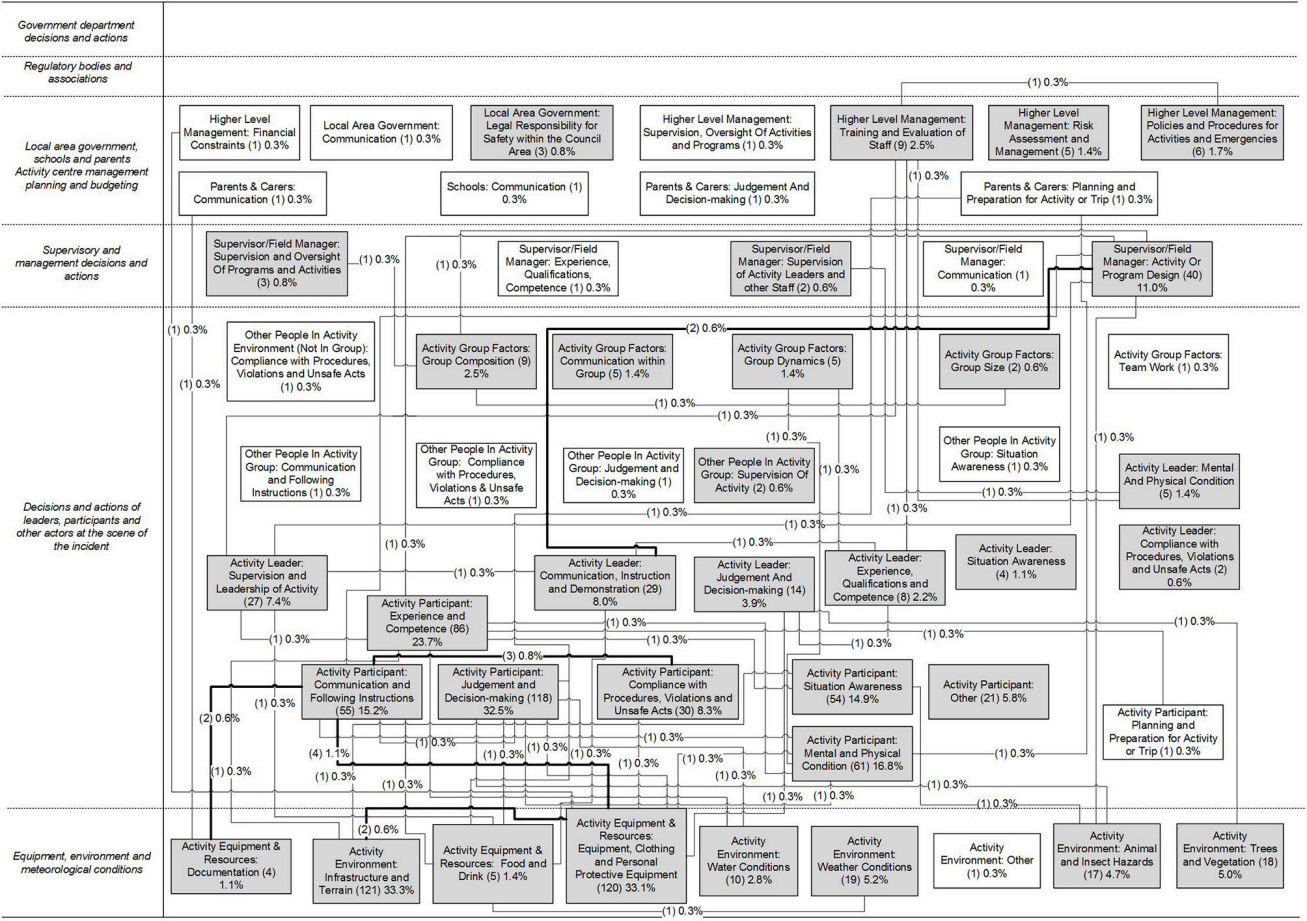
**Factors and relationships identified which contributed to injury-causing incidents**. Numbers in brackets indicate the number of incidents the factor or relationship was identified in. The total number of incidents analyzed was 364. Factors identified in more than one incident are shaded in gray, and relationships identified in more than one incident are bolded.

In total, 1020 incidents were reported, and 523 reports described the contributing factors and relationships involved in the incidents. These reports were analyzed by two members of the research team. This involved extracting a list of contributing factors and relationships between them from each report, discussing any discrepancies and reaching a consensus. The contributing factors and relationships were then classified using the scheme described in Figure 2. Summary AcciMaps were produced for each of the injury, illness and near miss data. This involved aggregating the contributing factor codes and the relationships between them across all the incidents within each type. The number of times the code and relationship appearing within the data were indicated on each AcciMap. Only the prevention strategies relating to the injury data are presented in this paper; Figure 4 presents the AcciMap analysis for this data.

A report was then produced with sections on the injury, illness and near miss data. Each section of the report included descriptive statistics (e.g., led outdoor activities associated with incidents, severity ratings, demographics of people involved), AcciMaps, tables describing the specific contributing factors and relationships underpinning the information presented in the AcciMaps, and text descriptions of the findings.

For the workshop, summaries of the results were produced for the injury, illness and near miss data. In addition, large print-outs of the AcciMaps were given to each group, as well as blank AcciMap templates (i.e., diagrams with the six AcciMap levels labeled). These were used to document the networks of prevention strategies generated during the workshop.

### Procedure

Two workshops were held; one in Brisbane and one in Melbourne, Australia. Prior to the workshops, participants were emailed the aims of the workshop and the incident data report. The report was provided to give participants time to read through the analysis in detail.

On arrival at the workshop, participants were introduced to the objectives of the session and provided written consent to take part in the study. Participants were then presented with information about Rasmussen's risk management framework and the AcciMap method, and introduced to Rasmussen's predictions regarding accident causation. They were then given a presentation on the key findings from the analysis of the injury, near miss and illness data, including an overview of the AcciMaps. They were given instructions on how to interpret the AcciMaps and data tables within the report and were given an example of why component-orientated prevention strategies might be unsuccessful. They were also provided with a simple example of a network of prevention strategies relating to the prevention of blisters, mapped onto an AcciMap template.

Next, participants partook in small group discussions, with each group led by a facilitator. These discussions were audio-recorded using a dictaphone. The discussions occurred in three rounds, each lasting approximately 45 min each. In the first round, participants considered the injury data, in the second round the illness data and in the third round the near miss data. Participants remained in the same small group for each round. There was a total of 7 groups across both workshops.

At the start of each round of discussion, participants were first asked to review the AcciMaps and data tables, and discuss the contributing factors. Where participants offered additional contributing factors that they believed from experience had a role in the events, these were documented by the facilitator. Participants were then encouraged to discuss potential prevention strategies and to consider how prevention strategies could be linked in a network or cluster of prevention strategies across the LOA system. Participants could choose whether to focus on developing prevention strategies to address specific issues identified in the data (e.g., burns resulting from cooking and campfires), or the total dataset. The reflection points were used either to prompt initial ideas or to refine ideas that were generated by participants. The facilitators documented the prevention strategies, and links between them, on the blank AcciMap templates. Each prevention strategy was described on the AcciMap in terms of the actors primarily responsible for implementation and the specific actions required (e.g., “National Parks: change camping permits to improve access to severe weather camping sites when required”). At the conclusion of the discussions, the facilitators presented each PreventiMap to the group, and made any additions or changes based on feedback.

### Data analysis

Due to space restrictions, only the prevention strategies relating to the injury data were analyzed for this paper.

The 7 PreventiMaps developed by the groups to address the key findings from the injury data were represented in Microsoft Visio. Each PreventiMap was reviewed and amended (to ensure clarity of description) by the facilitator who had originally documented it. Audio recordings were used when further information was needed to provide a more specific description of the prevention strategies. In addition, where appropriate, the facilitator created separate PreventiMaps to represent the specific goals their groups had discussed. This resulted in 10 PreventiMaps representing specific goals for incident prevention based on the injury data.

To identify similar prevention strategies across the groups, the PreventiMaps were coded using Nvivo 10. Each individual prevention strategy was coded into a theme based on: (1) the actors identified as responsible for implementation (e.g., Peak body); and (2) the specific actions required (e.g., lobby the government regarding the need to educate community on the benefits of LOA). A summary PreventiMap was then constructed by the researchers representing the prevention strategies that were identified by the workshop groups.

In addition, the 10 PreventiMaps representing specific goals for incident prevention were evaluated using the criteria presented in Table 2. The evaluation involved examining each PreventiMap, and giving a “Yes,” “Partial,” or “No” rating based on the criteria. “Yes” and “Partial” ratings had to be supported by examples, which were recorded in a table. The evaluation was initially conducted by the first author, and then validated by the second author. Any disagreements were resolved through discussion.

## Results

This section first presents an overall summary of all the prevention strategies identified by the workshop groups in relation to the injury data, as well as an example of a PreventiMap developed to address a specific goal. A summary of the findings from the evaluation is then presented. Note that throughout the results section “n” refers to the number of workshop groups (total *n* = 7).

### Description of prevention strategies

Based on the injury data, the workshop groups identified the following specific goals for incident prevention:

1. The prevention and management of Activity Leader fatigue (Group 1)
2. The prevention of burns during cooking activities (Group 2)
3. Improvement of participants' skills for outdoor activities (Group 3)
4. Improvement of reporting of pre-existing injuries (Group 3)
5. Ensuring that the difficulty of program matches participants' competence level (Group 4)
6. Improvement of communication around participant competence levels (Group 5)
7. Improvement of participants' physical literacy (Group 5)
8. Improvement of activity leaders' competencies around dynamic risk assessment (Group 6)
9. Professionalization of the career pathway for people in the LOA sector (Group 6)
10. Improvement of activity leaders' competencies for dealing with injuries (Group 7).

**Figure 5:**
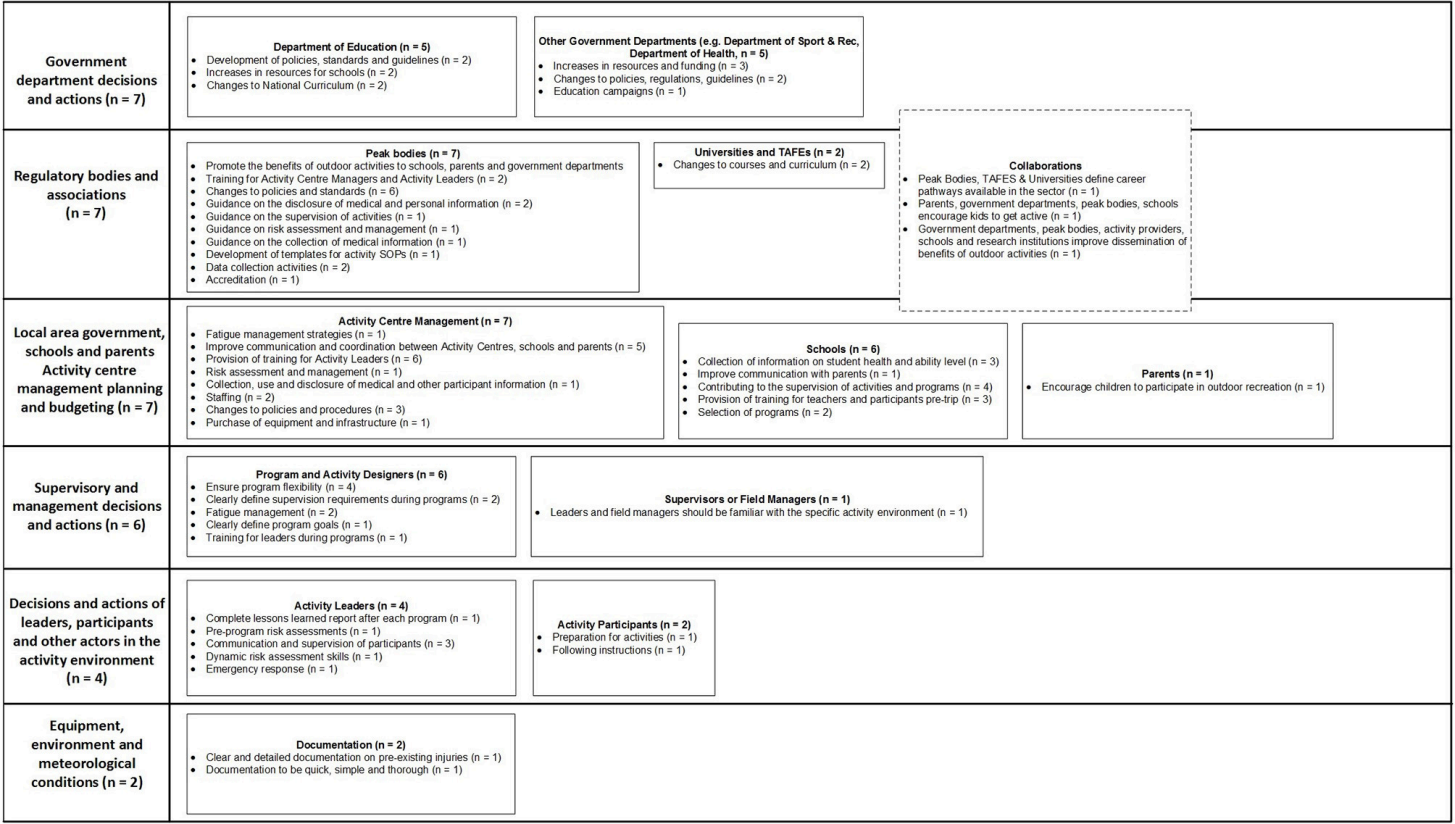
**Summary of the prevention strategies identified by workshop participants in relation to the injury data, presented according to the actors responsible for implementing the prevention strategy and the key themes**. Numbers in brackets indicate the number of workshop groups that identified the theme. The total number of workshop groups was 7.

Figure 5 shows a summary of all the prevention strategies identified to address these goals. Notably, prevention strategies were identified at all levels of the LOA system and in relation to all actors represented within the UPLOADS classification scheme. Some prevention strategies specifically addressed improving communication and collaboration between actors. The majority of prevention strategies focused on actions required at the second and third level of the framework. The actors most frequently identified as responsible for implementation were Peak bodies and Activity Center Management. The prevention strategy themes most frequently identified were “Peak bodies: Changes to policies and standards” (*n* = 6), “Activity Center: Improve communication and coordination between Activity Centers, schools and parents” (*n* = 5), and “Activity Center: Provision of training for Activity Leaders (*n* = 6).

All prevention strategies that were coded as “Peak bodies: Changes to policies and standards” focused on changes to the Adventure Activity Standards (AAS), which are voluntary safety guidelines for organizations conducting LOA. For example, to improve the quality of supervision during programs, Group 1 suggested that the AAS should “…incorporate Activity Leaders hours of work spent driving, active supervision and inactive supervision during programs,” while Group 2 suggested that the AAS should “…include supervision requirements and ratios around camp craft and camping.” Both prevention strategies were in response to the finding that “Activity Leader: Supervision and Leadership of Activity” and “Activity Leader: Communication, Instruction and Demonstration” were involved in just under 10% of all injury-causing incidents as shown in Figure 4.

The majority of prevention strategies coded as “Activity Center: Improve communication and coordination between Activity Centers, schools, and parents” focused on improving communication regarding participant experience, abilities and pre-existing injuries. For example, Group 2 suggested that Activity Centers should “improve communication with parents about child's previous experience outdoors,” while Group 6 suggested they should improve “…communication between schools and Activity Centers around participants health and abilities.” These prevention strategies were in response to the finding that many injury-causing incidents were caused by “Activity Participant: Experience and Competence” and “Activity Participant: Mental and Physical Condition,” which were identified in 24% and 17% of injury-causing incidents, respectively, as shown in Figure 4.

The prevention strategies coded as “Activity Center: Provision of training for Activity Leaders” addressed a range of weaknesses discussed in relation to Activity Leader skill sets. For example, Group 1 suggested that Activity Centers should “…provide soft skill training for co-leaders and distributed leadership,” while Group 4 suggested “…training for instructors to assist them to adapt program designs to suit the competence of the group.” Again, these prevention strategies were in response to a range of contributing factors relating to Activity Leaders supervision, competence and decision-making, as well as the incidents involving issues with Activity or Program design (identified as a contributing factor in 7% of injury-causing incidents, shown in Figure 4).

#### Example of a PreventiMap

**Figure 6:**
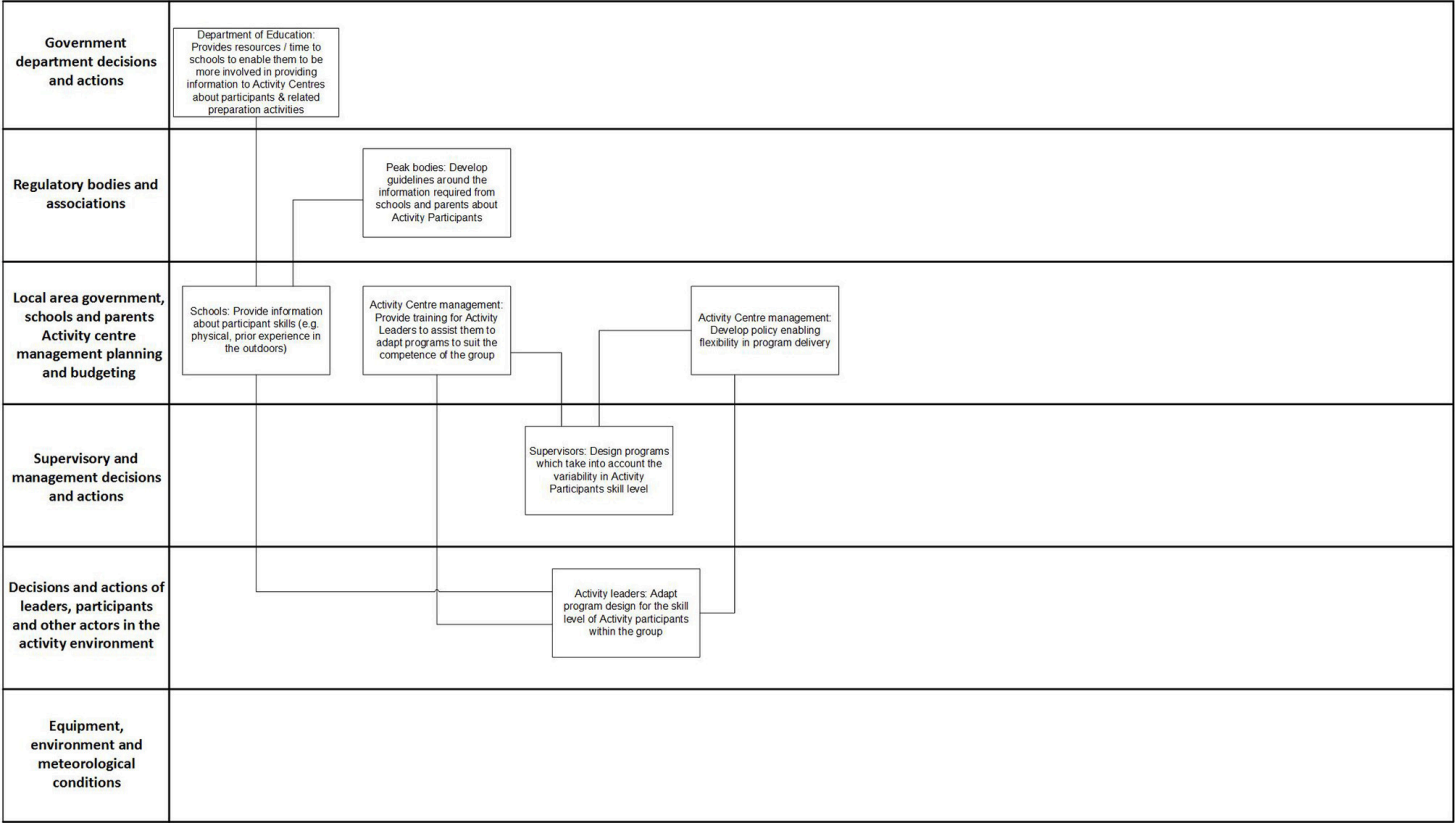
**PreventiMap developed by Group 4 to ensure that the difficulty of program matches participants' competence level**.

Figure 6 shows an example of the PreventiMaps developed by Group 4 to “ensure that the difficulty of the program matches participant skill levels.” This was in response to two of the most frequently identified contributing factors in injury-causing incidents: “Activity Participant: Experience and Competence” and “Activity Participant: Communication and Following Instructions.” These factors were identified in 24% and 15% of the injury incidents, respectively, and were highly interconnected to other factors on the AcciMap (see Figure 4). Workshop participants believed that many injuries occurred because program design did not adequately take into account Activity Participants' level of experience in the outdoors, and Activity Participants were ill prepared for the program (in terms of both physical literacy/fitness and equipment). Workshop participants discussed their perception that the skill level of participants had decreased over time, as children were less exposed to the outdoors and physical activity in their daily lives than previously.

The prevention strategies focus on improving communication between different actors within the system regarding participants' skills and implementing systems to increase the flexibility of program design. For example, workshop participants suggested that the Department of Education should provide more resources and time to enable schools to prepare participants for programs and gather information about their skills and abilities, which in turn, would enable schools to collect and provide information to Activity Centers on participants' competence. Activity Centers would then feed this information down into the development of programs. Workshop participants also suggested that Activity Leaders should be able to dynamically adapt programs to suit the skills of the group. They suggested that training on how to identify the skills of participants and adapt programs, as well as specific policies enabling flexibility in program delivery, would be needed to support Activity Leaders performing this function.

### Evaluation of PreventiMaps

The evaluation focused on the 10 PreventiMaps representing specific goals for incident prevention (described in Section Description of Prevention Strategies). The following sections present the findings in relation to the criteria, with selected examples to support the ratings. The PreventiMaps are referred to by the numbers shown in Table 3, which also summarizes the ratings from the evaluation. Table 4 summarizes the findings supporting the ratings for the first three evaluation criteria.

**Table 3:** **Summary of evaluation ratings for each PreventiMap representing specific goals for incident prevention**.

		**Criteria**							
**PreventiMap**	**Goal**	**1**	**2**	**3**	**4**	**5**	**6**	**7**	**8**
1	The prevention and management of Activity Leader fatigue	Yes	Yes	Yes	Partial	No	No	No	No
2	The prevention of burns during cooking activities	Yes	Yes	Yes	Partial	No	No	No	No
3	Improvement of participants' skills for outdoor activities	Yes	Yes	Yes	Yes	No	No	No	No
4	Improvement of reporting of pre-existing injuries	Yes	Yes	Yes	Yes	No	Yes	No	Yes
5	Ensuring that the difficulty of program matches participants competence level	Yes	Yes	Yes	Yes	No	No	No	No
6	Improvement of communication around participant competence levels	Yes	Yes	Yes	Yes	No	No	Partial	No
7	Improvement of participants' physical literacy	Yes	Yes	Yes	Partial	No	Partial	No	No
8	Improvement of activity leaders' competencies around dynamic risk assessment	Yes	Yes	Yes	Yes	No	Yes	No	No
9	Professionalization of the career pathway for people in the LOA sector	Yes	Yes	Yes	Yes	No	Partial	Partial	No
10	Improvement of activity leaders' competencies for dealing with injuries	Yes	Yes	Yes	Yes	No	Yes	Partial	Yes

**Table 4:** **Summary of the findings supporting the ratings for the first three evaluation criteria**.

	**Criterion 1**											**Criterion 2**						**Criterion 3**	
	**Actors identified as responsible for implementation**											**LOA system levels required to change**							
**PreventiMap**	**Government**	**Department of Education**	**Universities/TAFEs**	**Research groups**	**Peak bodies**	**Schools**	**Parents**	**Activity Center Management**	**Supervisors/Field Managers**	**Activity Leaders**	**Activity Participants**	**1**	**2**	**3**	**4**	**5**	**6**	**No. of prevention strategies**	**No. of relationships**
1		X	X		X	X		X	X			X	X	X	X			13	15
2		X	X		X	X		X	X	X		X	X	X	X	X		13	8
3	X					X	X	X	X	X	X	X		X	X	X		9	7
4		X			X		X	X				X	X	X			X	6	6
5		X			X	X		X		X		X	X	X		X		7	7
6	X	X			X	X	X	X				X	X	X				16	20
7	X	X			X	X	X					X	X	X				6	9
8	X		X		X			X				X	X	X				9	9
9			X	X	X			X				X	X	X				6	4
10					X	X		X	X	X			X	X	X	X		11	19

*LOA system levels: 1, Government department decisions and actions; 2, Regulatory bodies and associations; 3, Local area government, schools and parents, Activity Center management planning and budgeting; 4, Supervisory and management decisions and actions; 5, Decisions and actions of leaders, participants and other actors in the activity environment; 6, Equipment, environment and meteorological conditions*.

#### Criterion 1: the prevention strategies require actions and decisions from multiple actors (at least three)

All 10 PreventiMaps met this criterion. The PreventiMaps identified between 4 and 7 actors responsible for implementation. The actors most frequently identified as responsible were Peak bodies and Activity Center Management. While many of the contributing factors in the incident data related to Activity Participants, only one prevention strategy identified Activity Participants as playing a role in implementation.

#### Criterion 2: the prevention strategies require changes at multiple levels of the system (at least three)

All 10 PreventiMaps met this criterion. The PreventiMaps required changes to 3–5 system levels. All PreventiMaps required changes at the third level of the framework, and overall, they tended to focus on changes at the three highest levels of the system.

#### Criterion 3: multiple interdependent prevention strategies are identified to address the specified goal (at least three). these include mechanisms to support the implementation of prevention strategies within and across levels

All 10 PreventiMaps met this criterion. The PreventiMaps described between 6 and 16 prevention strategies, and 4–20 relationships.

Most of the mechanisms identified to support implementation occurred across levels. For example, a number of prevention strategies at the higher levels were identified to support the prevention strategy: “Activity Leaders adapt program design for their group,” including: flexibility is included in program design; Activity Centers provide training on how to adapt programs to suit competence levels; and Activity Centers develop a policy allowing Activity Leaders to change the delivery of programs (PreventiMap 5).

PreventiMap 3 included examples of across level support mechanisms. The prevention strategy “Activity Centers and Schools improve communication with parents around participant capabilities” was supported within the level by: schools improve briefing to parents around required levels of competence; and Activity Centers develop key descriptors of competence related to different types of activities.

#### Criterion 4: the prevention strategies support the flow of information from actors across and within system levels

Seven of the PreventiMaps fully met this criterion, and three partially met this criterion.

The PreventiMaps that fully met this criterion included prevention strategies to improve the flow of information between actors both across and within levels. For example, PreventiMap 8 included prevention strategies to improve communication across and within levels regarding risk assessments. Specifically, workshop participants identified the prevention strategy “Peak bodies provide opportunities to talk with Activity Centers about risk assessment and share experiences” to improve across level communication, while “Activity providers to provide risk assessments to parents, and consent forms are signed based on this information” was identified to improve within level communication.

The PreventiMaps that partially met this criterion only included prevention strategies that increased the flow of information in a specific direction. For example, PreventiMap 7 only targeted the flow of information between Level 1 and Level 2 of the LOA system (e.g., “Peak bodies to lobby government to establish independent body on physical literacy”).

#### Criterion 5: the prevention strategies improve feedback processes to actors regarding the impact of their decisions and actions

None of the PreventiMaps met this criterion. During the evaluation, it was noted that many of the PreventiMaps failed to identify mechanisms to monitor the impact of changes to regulations, policies and procedures. For example, although PreventiMap 1 describes a range of regulations, policies, and programs to prevent Activity Leader fatigue, no mechanism was identified for monitoring actual levels of fatigue.

#### Criterion 6: the prevention strategies provide mechanisms for actors at the higher levels to identify or monitor changes to work practices at the frontline of operation

Three of the PreventiMaps fully met this criterion, and two met it partially.

The PreventiMaps that fully met this criterion included prevention strategies to monitor changes to Activity Leader work practices. For example, PreventiMap 10 included a prevention strategy specifying that Activity Leaders should receive training on “understanding and identifying complexities of mental and physical health issues.” To monitor the impact of this program, it was proposed that Activity Centers should conduct “regular appraisals by peers and management to assess performance strengths and weaknesses to guide additional training.”

The PreventiMaps that partially met this criterion only implied avenues for monitoring changes to work practices at the frontline of operation. For example, PreventiMap 7 proposed that the government should provide more funding for school outdoor education programs, and change the school curriculum to include outdoor education. Potentially, government departments would monitor the take up of this funding and the implementation of changes to school curriculum; however, this was not explicitly specified by participants.

#### Criterion 7: the prevention strategies provide mechanisms for monitoring changes to work practices for actors at the higher levels of the system

None of the PreventiMaps fully met this criterion, and three met it partially.

The PreventiMaps that partially met this criterion only implied avenues for monitoring changes to work practices at the higher levels of the system. For example, PreventiMap 7 included a relationship between “Peak bodies to lobby government to establish independent body on physical literacy” and “Government to increase funding for outdoor education programs.” Potentially, the Peak bodies would monitor changes to funding at the government level, although this is not explicit. Similarly, PreventiMap 10 specified that “Activity Centres should set guidelines around the required number of permanent staff” to address the issues identified with causal staff lacking relevant knowledge and training. This prevention strategy would potentially prevent Activity Centers from hiring more causal staff in response to financial pressures.

#### Criterion 8: the prevention strategies include mechanisms for monitoring whether the implementation of risk control measures are degrading over time

Two of the PreventiMaps met this criterion. For example, the goal of PreventiMap 4 was to improve the reporting of pre-existing injuries. It was proposed that “data on incidents rates are made available on the websites of Peak bodies.” This provides a way of monitoring whether the risk control measures associated with reporting pre-existing are eroding over time at an industry level. Similarly, the goal of PreventiMap 10 was to improve activity leaders' competencies for dealing with injuries. It was proposed that activity leaders should receive “Regular appraisals by peers and management to assess performance.” This provides a way for organizations to monitor whether the risk control measures associated with dealing with pre-existing injuries are eroding over time.

In relation to this criterion, it was noted during the evaluation that some of the prevention strategies proposed might have the unintended consequence of eroding risk control measures over time. For example, PreventiMap 5 focused on increasing the flexibility of the delivery of programs, with the expectation that Activity Leaders would alter programs to match Activity Participants level of competence. However, Activity Leaders might become more focused on altering programs than ensuring that existing risk controls are maintained. In addition, altering programs might unintentionally result in new hazards. No prevention strategies were proposed to address these potential consequences.

## Discussion

The aims of this article were to describe the prevention strategies that were developed by applying a systems thinking approach during the design process, and to evaluate the extent to which they addressed the systemic causes of accidents as defined by Rasmussen's risk management framework. Using a systems thinking-based design process, workshop groups identified a range of specific goals for incident prevention from the injury data. To address these goals, PreventiMaps were developed representing prevention strategies requiring actions from all actors, across all levels of the system. All of the PreventiMaps required actions at the higher levels of the system, and only a few focused on the immediate context of LOA delivery. Prevention strategies involving actions at the frontline of system operation (e.g., Activity Leaders should adapt programs to suit participants capabilities) were supported by changes to policies, training and regulation. The subsequent evaluation of the PreventiMaps revealed that all of them addressed the three core principles of the systems approach (Criteria 1, 2, and 3), and the majority proposed prevention strategies for improving vertical integration (Criterion 4). However, overall the PreventiMaps tended to focus on top-down controls, rather than bottom-up feedback and monitoring of work practices. Therefore, the majority of the PreventiMaps failed to address Rasmussen's predictions regarding the migration of work practices over time and the erosion of risk control measures (Criterion 5–8). Overall, the evaluation shows that the design process was partially successful in helping practitioners to translate incident data into prevention strategies that address the systemic causes of accidents, and highlights areas for improvement in the design process.

The findings from this study will be used to improve the design process in a number of ways. First, the findings indicate that the reflection points need to be refined to focus practitioners more on identifying ways to monitor behavior and decision-making at the frontline of system operation, and designing feedback mechanisms to support decisions at the higher levels of the system. To support this aspect of the design process, it might be helpful to identify specific incidents from the LOA data where monitoring and feedback processes have failed, along with examples of successful monitoring and feedback processes used in other safety-critical domains. Second, the design process resulted in many, often overlapping, specific goals for incident prevention and prevention strategies. A further phase in the design process is required to refine and select specific goals and prevention strategies. This will require the development of further evaluation criteria to assist this decision-making process.

The approach used to design prevention strategies in this paper resulted in different outputs to the component-orientated approaches described in the literature (Johnson, 2003; Hollnagel, 2008; Lundberg et al., 2009, 2010; Rollenhagen, 2011). For example, based on an analysis of investigation manuals, Johnson (2003) describes four general approaches that are used by organizations to generate possible prevention strategies: the perfectibility approach; the heuristic approach; navigational techniques; and accident prevention models such as Haddon's (1980; see pp. 565–590 of Johnson, 2003, for a description and extensive discussion of the strengths and weaknesses of each approach). The key difference between the approaches is the type of “fixes” that are deemed appropriate or effective. However, all of the approaches focus on developing a list of prevention strategies; each item on the list is intended to address a specific component of the problem identified in the incident analysis. No consideration is given to the relationships between prevention strategies or the interactions between them once implemented. The approach used in this study allowed participants to understand the interdependencies between the solutions they were proposing, and to identify the mechanisms needed to support implementation across the system.

It should be acknowledged that the group problem solving approach to designing prevention strategies is not novel, although it does not appear to be consistently used across industries (Lundberg et al., 2010; Rollenhagen et al., 2010; Rollenhagen, 2011). In the context of Swedish nuclear power plants, Rollenhagen (2011) notes that problem solving groups that include representatives from the whole system of interest are perceived as more successful in identifying more effective prevention strategies. He attributes the perception of success to increasing actors understanding of the system functions they do not directly influence, and of the consequences of their decisions for other functions. The key difference between earlier studies and the current study is the boundary on the “system of interest.” In this study, the system included actors outside the context of the organization (e.g., Peak bodies, WHS regulators, and government departments). These actors had a detailed understanding of the guidelines, regulations, government policies, programs, and funding influencing LOA provision. This knowledge may have helped representatives from LOA providers think outside the “silo” of their organization, and consider the sector as a whole.

An unaddressed question from this study is the practicality of the prevention strategies proposed and whether the prevention strategies are likely to be implemented by the sector. For example, resource constraints are typically a significant factor that moderate the success of the prevention strategies proposed in response to incidents (Lundberg et al., 2010). The next phase of the research program involves inviting the whole sector to evaluate of the feasibility of the prevention strategies. However, it is acknowledged that even if the prevention strategies are favorably assessed by the sector, there are many factors that will influence their implementation (e.g., Pidgeon and O'Leary, 2000; Lundberg et al., 2010, 2012; Ramanujam and Goodman, 2011; Le Coze, 2013; Vastveit et al., 2015). A direction for future research is to chart the barriers to implementing system reforms that exists within the LOA sector.

The limitations of the study should be acknowledged, which also present some directions for future research. One significant limitation of the present study is a lack of comparison groups. For example, until the implementation of UPLOADS, the sector did not have good quality incident data to focus their preventative efforts (Goode et al., 2014a, 2015; Salmon et al., 2016a). Therefore, the same prevention strategies may have been identified based on the incident data analysis, without the design process. However, it seems unlikely that the networks of prevention strategies would have been generated without the design process or application of the AcciMap technique. To address this issue, the authors plan to conduct controlled trials to compare the design process against unstructured group brainstorming sessions. In addition to evaluating the extent to which the proposed prevention strategies address the systemic causes of accidents, a scale developed by Jacobsson's et al. (2011) will be used to evaluate their potential effectiveness. This scale evaluates the effectiveness of prevention strategies on three dimensions: geographical application, degree of organizational learning, and time. More effective prevention strategies are those that apply across the organization, target the redesign of organizational systems, and involve plans for long term maintenance. The authors also plan to evaluate potential improvements in prevention strategies, and modifications to the design process, as the process is implemented in an organization over an extended period of time. Future studies are also required to determine the training requirements of implementing the design process in an organization to ensure that it produces valid outputs (Stanton and Stevenage, 1998; Stanton, 2016).

A second limitation of this study was that two important actors were missing from the workshops—activity participants and the parents of children involved in the activities—the LOA sector's “consumers.” These actors may have a different view on the factors that would encourage them to play a more active role in managing risk. Accordingly, it is recommended that they are represented at future workshops.

In conclusion, the approach applied in this study allowed practitioners to create networks of prevention strategies designed to address the conditions at the higher levels of the LOA system. This approach to prevention strategy design is not only novel for the LOA sector, but across the safety critical domains. To the authors' knowledge, this study is the first reported to apply Rasmussen's Risk Management Framework and AcciMap technique to incident data collection, incident analysis and prevention strategy design, all as part of an integrated process. Most importantly, the prevention strategies were designed by the actors within the system of interest, rather than by researchers studying the system. We encourage further applications of the approach, and future research should consider how these methods might apply to the next steps in the learning cycle (Jacobsson et al., 2012): decision-making, implementation and follow-up.

## Author contributions

NG Conceived and designed of the study, organized the data collection, facilitated the workshops, conducted the evaluation, analyzed the data and wrote the manuscript. GR Contributed to the conception and design of the study, facilitated the workshops, conducted the evaluation, contributed to writing and revising the manuscript. MV Contributing to organizing the data collection, facilitated the workshops, transcribed the data, contributed to analyzing the data, and contributed to revising the manuscript. AC Facilitated the workshops, transcribed the data, contributed to analyzing the data, and contributed to revising the manuscript. PS Conceived and designed of the study, facilitated the workshops, contributed to the evaluation, and contributed to writing and revising the manuscript.

### Conflict of interest statement

The authors declare that the research was conducted in the absence of any commercial or financial relationships that could be construed as a potential conflict of interest.

## Footnotes

^1^The term “prevention strategies” is used interchangeably in the literature with other terms such as “prevention strategies,” “prevention activities,” “recommendations,” “remedial actions,” “corrective actions,” “countermeasures” and “interventions.”

## References

[B1] Branford K. (2011). Seeing the big picture of mishaps: applying the AcciMap approach to analyze system accidents. Aviat. Psychol. Appl. Hum. Factors 1, 31–37. 10.1027/2192-0923/a00005

[B2] Brookes A. (2003). Outdoor education fatalities in Australia 1960-2002. Part 2. Contributing circumstances: supervision, first aid, and rescue. Aus. J. Outdoor Educ. 7, 34–42. Available online at: http://www.latrobe.edu.au/education/downloads/brookes_a_OE-Fatalities2.pdf (Accessed December 15, 2016).

[B3] Brookes A. (2004). Outdoor education fatalities in Australia 1960-2002. Part 3. Environmental circumstances. Aus. J. Outdoor Educ. 8, 44–56. Available online at: http://www.latrobe.edu.au/education/downloads/brookes_a_OE-Fatalities3.pdf (Accessed December 15, 2016).

[B4] Brookes A. Smith M. Corkill B (2009). Report to the Trustees of the Sir Edmund Hillary Outdoor Pursuit Centre of New Zealand: Mangatepopo Gorge Incident. Available online at: http://www.hillaryoutdoors.co.nz/newsite/wp-content/uploads/2013/06/091015-IRT-OPC_-Report.pdf

[B5] Carroll J. S. Fahlbruch B. (2011). The gift of failure: new approaches to analyzing and learning from events and near-misses. Honoring the contributions of Bernhard Wilpert. Safety Sci. 49, 1–4. 10.1016/j.ssci.2010.03.005

[B6] Cassano-Piche A. L. Vicente K. J. Jamieson G. A. (2009). A test of Rasmussen's risk management framework in the food safety domain: BSE in the UK. Theor. Issues Ergon. Sci. 10, 283–304. 10.1080/14639220802059232

[B7] Curtis R (1995). OA Guide to Outdoor Safety Management. Available online at: https://www.princeton.edu/~oa/safety/safeman.html

[B8] Davenport C. J (2010). Mangatepopo Coroners Report. Auckland. Available online at: http://outdoorcouncil.asn.au/doc/Coroners_Report_OPC.pdf

[B9] Dekker S. (2011). Drift Into Failure: From Hunting Broken Components to Understanding Complex Systems. Boca Raton, FL: CRC Press, Taylor & Francis Group.

[B10] Dekker S. Cilliers P. Hofmeyr J. H. (2011). The complexity of failure: implications of complexity theory for safety investigations. Saf. Sci. 49, 939–945. 10.1016/j.ssci.2011.01.008

[B11] Department of Health (2006). Safety First: A Report for Patients, Clinicians and Healthcare Managers. Available online at: http://webarchive.nationalarchives.gov.uk/20130107105354/http://www.dh.gov.uk/prod_consum_dh/groups/dh_digitalassets/@dh/@en/documents/digitalasset/dh_064159.pdf

[B12] Drupsteen L. Groeneweg J. Zwetsloot G. (2013a). Critical steps in learning from incidents: using learning potential in the process from reporting an incident to accident prevention. Int. J. Occup. Saf. Ergon. 19, 63–77. 10.1080/10803548.2013.11076966 23498711

[B13] Drupsteen L. Groeneweg J. Zwetsloot G. (2013b). Critical steps in learning from incidents: using learning potential in the process from reporting an incident to accident prevention. Int. J. Occupat. Saf. Ergon. 19, 63–77. 10.1080/10803548.2013.11076966 23498711

[B14] Drupsteen L. Hasle P. (2014). Why do organizations not learn from incidents? Bottlenecks, causes and conditions for a failure to effectively learn. Accid. Anal. Prev. 72, 351–358. 10.1016/j.aap.2014.07.027 25118127

[B15] Goode N. Finch C. Cassell E. Lenne M. G. Salmon P. M. (2014a). What would you like? Identifying the required characteristics of an industry-wide incident reporting and learning system for the led outdoor activity sector. Aus. J. Outdoor Educ. 17, 2–15. Available online at: http://research.usc.edu.au/vital/access/services/Download/usc:12724/SOURCE2 (Accessed December 16, 2016).

[B16] Goode N. Salmon P. M. Lenne M. Finch C. F. (2014b). A test of a systems theory-based incident coding taxonomy for risk managers, in Advances in Safety Management and Human Factors, Vol. 10, *Advances in Human Factors and Ergonomics 2014*, eds Arezes P. Carvalho P., 5098–5108.

[B17] Goode N. Salmon P. M. Lenne M. G. Taylor N. Z. Finch C. F. (2015). The UPLOADS Project: development of an Australian National Incident Dataset for Led Outdoor Activities. Wilderness Environ. Med. 26, 574–576. 10.1016/j.wem.2015.04.006 26141920

[B18] Haddon W. (1980). Advances in the epidemiology of injuries as a basis for public policy. Public Health Rep. 95, 411–421. Retrieved from: http://www.jstor.org/stable/4596353 7422807 PMC1422748

[B19] Hollnagel E. (2004). Barriers and Accident Prevention. Aldershot: Ashgate.

[B20] Hollnagel E. (2008). Risk + barriers = safety? Saf. Sci. 46, 221–229. 10.1016/j.ssci.2007.06.028

[B21] Hollnagel E. (2012). FRAM: the Functional Resonance Analysis Method: Modelling Complex Socio-Technical Systems. Aldershot: Ashgate Publishing, Ltd.

[B22] Jenkins D. P. Salmon P. M. Stanton N. A. Walker G. H. (2010). A systemic approach to accident analysis: a case study of the Stockwell shooting. Ergonomics 53, 1–17. 10.1080/00140130903311625 20069477

[B23] Jacobsson A. Ek A. Akselsson R. (2011). Method for evaluating learning from incidents using the idea of “level of learning”. J. Loss Prev. Process Industries 24, 333–343. 10.1016/j.jlp.2011.01.011

[B24] Jacobsson A. Ek A. Akselsson R. (2012). Learning from incidents – A method for assessing the effectiveness of the learning cycle. J. Loss Prev. Process Industries 25, 561–570. 10.1016/j.jlp.2011.12.013

[B25] Jacobsson A. Sales J. Mushtaq F. (2010). Underlying causes and level of learning from accidents reported to the MARS database. J. Loss Prev. Process Industries 23, 39–45. 10.1016/j.jlp.2009.05.002

[B26] Johnson C. W. (2003). Failure in Safety-Critical Systems: A Handbook of Incident and Accident Reporting. University of Glasgow.

[B27] Johnson C. W. Muniz de Almeida I. (2008). Extending the borders of accident investigation: applying novel analysis techniques to the loss of the Brazilian space launch vehicle VLS-1 V03. Saf. Sci. 46, 38–53. 10.1016/j.ssci.2006.05.007

[B28] Katsakiori P. Sakellaropoulos G. Manatakis E. (2009). Towards an evaluation of accident investigation methods in terms of their alignment with accident causation models. Saf. Sci. 47, 1007–1015. 10.1016/j.ssci.2008.11.002

[B29] Kirwan B. (2011). Incident reduction and risk migration. Saf. Sci. 49, 11–20. 10.1016/j.ssci.2010.03.007

[B30] Le Coze J. (2013). What have we learned about learning from accidents? Post-disasters reflections. Saf. Sci. 51, 441–453. 10.1016/j.ssci.2012.07.007

[B31] Leveson N. (2011). Applying systems thinking to analyze and learn from events. Saf. Sci. 49, 55–64. 10.1016/j.ssci.2009.12.021

[B32] Lindberg A. K. Hansson S. O. Rollenhagen C. (2010). Learning from accidents – What more do we need to know? Saf. Sci. 48, 714–721. 10.1016/j.ssci.2010.02.004

[B33] Lundberg J. Rollenhagen C. Hollnagel E. (2009). What-You-Look-For-Is-What-You-Find – The consequences of underlying accident models in eight accident investigation manuals. Saf. Sci. 47, 1297–1311. 10.1016/j.ssci.2009.01.004

[B34] Lundberg J. Rollenhagen C. Hollnagel E. (2010). What you find is not always what you fix—How other aspects than causes of accidents decide recommendations for remedial actions. Accid. Anal. Prev. 42, 2132–2139. 10.1016/j.aap.2010.07.003 20728672

[B35] Lundberg J. Rollenhagen C. Hollnagel E. Rankin A. (2012). Strategies for dealing with resistance to recommendations from accident investigations. Accid. Anal. Prev. 45, 455–467. 10.1016/j.aap.2011.08.014 22269530

[B36] Newnam S. Goode N. (2015). Do not blame the driver: a systems analysis of the causes of road freight crashes. Accid. Anal. Prev. 76, 141–151. 10.1016/j.aap.2015.01.016 25645163

[B37] Nielsen K. J. Carstensen O. Rasmussen K. (2006). The prevention of occupational injuries in two industrial plants using an incident reporting scheme. J. Saf. Res. 37, 479–486. 10.1016/j.jsr.2006.06.005 17123544

[B38] Pidgeon N. O'Leary M. (2000). Man-made disasters: why technology and organizations (sometimes) fail. Saf. Sci. 34, 15–30. 10.1016/S0925-7535(00)00004-7

[B39] Pless B. (2008). Surveillance alone is not the answer. Inj. Prev. 14, 220–222. 10.1136/ip.2008.019273 18676778

[B40] Ramanujam R. Goodman P. S. (2011). The challenge of collective learning from event analysis. Saf. Sci. 49, 83–89. 10.1016/j.ssci.2010.03.019

[B41] Rasmussen J. (1997). Risk management in a dynamic society: a modelling problem. Saf. Sci. 27, 183–213. 10.1016/S0925-7535(97)00052-0

[B42] Read G. J. M. Salmon P. M. Lenné M. G. Jenkins D. P. (2015). Designing a ticket to ride with the Cognitive Work Analysis Design Toolkit. Ergonomics 58, 1266–1286. 10.1080/00140139.2015.1013576 25805238

[B43] Major Accidents Reporting System (2012). Available online at: https://emars.jrc.ec.europa.eu/

[B44] Aviation Safety Reporting System (2015). ASRS Program Briefing. Available online at: http://asrs.arc.nasa.gov/docs/ASRS_ProgramBriefing2015.pdf

[B45] Rollenhagen C. (2011). Event investigations at nuclear power plants in Sweden: reflections about a method and some associated practices. Saf. Sci. 49, 21–26. 10.1016/j.ssci.2009.12.012

[B46] Rollenhagen C. Westerlund J. Lundberg J. Hollnagel E. (2010). The context and habits of accident investigation practices: a study of 108 Swedish investigators. Saf. Sci. 48, 859–867. 10.1016/j.ssci.2010.04.001

[B47] Salmon P. M. Cornelissen M. Trotter M. J. (2012). Systems-based accident analysis methods: a comparison of Accimap, HFACS, and STAMP. Saf. Sci. 50, 1158–1170. 10.1016/j.ssci.2011.11.009

[B48] Salmon P. M. Goode N. Archer F. Spencer C. McArdle D. McClure R. J. (2014a). A systems approach to examining disaster response: using Accimap to describe the factors influencing bushfire response. Saf. Sci. 70, 114–122. 10.1016/j.ssci.2014.05.003

[B49] Salmon P. M. Goode N. Lenné M. G. Finch C. F. Cassell E. (2014b). Injury causation in the great outdoors: a systems analysis of led outdoor activity injury incidents. Accid. Anal. Prev. 63, 111–120. 10.1016/j.aap.2013.10.019 24284079

[B50] Salmon P. M. Goode N. Taylor N. Z. Lenne M. G. Dallat C. Finch C. F. (2016a). Rasmussen's legacy in the great outdoors: a new incident reporting and learning system for led outdoor activities. Appl. Ergon. 59, 637–648. 10.1016/j.apergo.2015.07.017 26897478

[B51] Salmon P. M. Read G. Stanton N. A. Lenné M. (2013). The Crash at Kerang: investigating systemic and psychological factors leading to unintentional non-compliance at rail level crossings. Accid. Anal. Prev. 50, 1278–1288. 10.1016/j.aap.2012.09.029 23122328

[B52] Salmon P. M. Walker G. H. Read G. J. M. Goode N. Stanton N. A. (2016b). Fitting methods to paradigms: are ergonomics methods fit for systems thinking? Ergonomics 22, 1–12. 10.1080/00140139.2015.1103385 26799501

[B53] Salmon P. M. Williamson A. Lenne M. Mitsopoulos-Rubens E. Rudin-Brown C. M. (2010). Systems-based accident analysis in the led outdoor activity domain: application and evaluation of a risk management framework. Ergonomics 53, 927–939. 10.1080/00140139.2010.489966 20658387

[B54] Stanton N. A. (2016). On the reliability and validity of, and training in, ergonomics methods: a challenge revisited. Theor. Issues Ergon. Sci. 17, 345–353. 10.1080/1463922X.2015.1117688

[B55] Stanton N. A. Stevenage S. V. (1998). Learning to predict human error: issues of acceptability, reliability and validity. Ergonomics 41, 1737–1756. 10.1080/001401398186162 9819584

[B56] Svedung I. Rasmussen J. (2002). Graphic representation of accidentscenarios: mapping system structure and the causation of accidents. Saf. Sci. 40, 397–417. 10.1016/S0925-7535(00)00036-9

[B57] Taylor N. Z. Goode N. Salmon P. M. Lenne M. G. Finch C. F. (2015a). Inter-rater reliability of a causal factor taxonomy that uses systems theory designed for the outdoor domain, in Paper Presented at the AHFE2015, 1st International Conference on Human Factors in Sports and Outdoor Recreation (Las Vegas, NV).

[B58] Taylor N. Z. Goode N. Salmon P. M. Lenne M. G. Finch C. F. (2015b). Which code is it? Inter-rater reliability of systems theory-based causal factor taxonomy for the outdoor sector, in Paper Presented at the 19th Triennial Congress of the International Ergonomics Association (Melbourne, VIC).

[B59] Underwood P. Waterson P. (2014). Systems thinking, the Swiss Cheese Model and accident analysis: a comparative systemic analysis of the Grayrigg train derailment using the ATSB, AcciMap and STAMP models. Accid. Anal. Prev. 68, 75–94. 10.1016/j.aap.2013.07.027 23973170

[B60] van Mulken M. Clacy A. Grant E. Goode N. Finch C. F. Stevens E. (2016). The UPLOADS National Incident Dataset The First Twelve Months: 1st June 2014 to 31st May 2015. Maroochydore: Centre for Human Factors and Sociotechnical Systems https://uploadsproject.files.wordpress.com/2016/03/first-12-month-report_final.pdf (Accessed December 15, 2016).

[B61] Vastveit K. R. Boin A. Njå O. (2015). Learning from incidents: practices at a Scandinavian refinery. Saf. Sci. 79, 80–87. 10.1016/j.ssci.2015.05.001

[B62] Vicente K. J. (1999). Cognitive Work Analysis: Toward Safe, Productive, and Healthy Computer-Based Work. CRC Press.

[B63] Vincent C. (2004). Analysis of clinical incidents: a window on the system not a search for root causes. Qual. Saf. Health Care 13, 242–243. 10.1136/qshc.2004.010454 15289620 PMC1743862

